# Chemical Profiling, Ampicillin Interaction Patterns, and Exploratory Molecular Docking of *Lauraceae* Essential Oils

**DOI:** 10.3390/ijms27031447

**Published:** 2026-01-31

**Authors:** Anca Hulea, Florin Imbrea, Doris Floares (Oarga), Iuliana Popescu, Mukhtar Adeiza Suleiman, Calin Hulea, Ilinca Merima Imbrea, Alina-Georgeta Neacșu, Marinel Horablaga, Cosmin Alin Popescu, Diana Obistioiu

**Affiliations:** 1 Faculty of Agriculture, University of Life Sciences “King Michael I” from Timisoara, Calea Aradului 119, 300645 Timisoara, Romania; anca.hulea@usvt.ro (A.H.); doris.oarga@usvt.ro (D.F.); iuliana_popescu@usvt.ro (I.P.); alinaneacsu@usvt.ro (A.-G.N.); marinel_horablaga@usvt.ro (M.H.); cosmin_popescu@usvt.ro (C.A.P.); dianaobistioiu@usvt.ro (D.O.); 2 Department of Biochemistry, Faculty of Life Science, Ahmadu Bello University, Zaria 810107, Nigeria; masuleiman@abu.edu.ng; 3 Faculty of Veterinary Medicine, University of Life Sciences “King Michael I” from Timisoara, Calea Aradului 119, 300645 Timisoara, Romania; calin.hulea@usvt.ro; 4 Faculty of Engineering and Applied Technologies, University of Life Sciences “King Michael I” from Timisoara, Calea Aradului 119, 300645 Timisoara, Romania; ilinca_imbrea@usvt.ro; 5 Agricultural Research and Development Station Lovrin, Principala Street No. 200, 307260 Lovrin, Romania

**Keywords:** *Lauraceae* essential oils, *Litsea cubeba*, *Laurus nobilis*, *Cryptocarya agathophylla*, bacterial inhibition percentage, molecular docking

## Abstract

This study compares the chemical composition, antimicrobial effects, and antibiotic-potentiating capacity of three *Lauraceae* essential oils (EO): *Cryptocarya agathophylla* (CAEO), *Litsea cubeba* (LCEO), and *Laurus nobilis* (LNEO). Gas chromatography–mass spectrometry (GC–MS) analysis revealed distinct chemotypes: CAEO and LCEO were dominated by oxygenated monoterpenes, while LNEO contained the highest levels of monoterpene hydrocarbons. Antibacterial testing against nine bacterial strains showed strain-dependent growth suppression trends, while true minimum inhibitory concentrations (MICs) were reached only in selected cases. EO–ampicillin interactions were evaluated using MIC-based checkerboard criteria, whereas OD-derived inhibition parameters were used exclusively to describe sub-MIC potentiation trends. In combination assays, LNEO exhibited the most pronounced potentiating effects against *Streptococcus pyogenes*, *Shigella flexneri*, and *Haemophilus influenzae*, while CAEO and LCEO showed moderate or strain-dependent enhancement. Hierarchical clustering highlighted distinct oil- and strain-specific interaction profiles. Overall, although CAEO displayed stronger intrinsic antibacterial effects when tested alone, LNEO emerged as the most effective potentiator of ampicillin activity in a strain-dependent manner. The effects of the major compounds identified in the *Lauraceae* EO were assessed in silico against protein targets of some microorganisms using the AutoDock software version 4.2.6. The docking scores revealed binding affinities of the bioactive compounds towards Dpr protein (4.3–5.8 kcal/mol), DNA gyrase (4.7–7.1 kcal/mol), mono- diacylglycerol lipase (4.4–6.2 kcal/mol), CYP51 (5.8–8.0 kcal/mol), phage-encoded quorum sensing anti-activator (5.8–8.0 kcal/mol) and Chondroitin ABC lyase I (4.8–6.3 kcal/mol). Two (2) hit compounds (α-Citral, β-Citral) were finely defined by strong hydrophobic and hydrophilic interactions with the bacterial and fungal protein targets, respectively.

## 1. Introduction

The *Lauraceae* family is a group of pantropical plants that includes approximately 55 genera and 3000 species of trees and shrubs. Some of the species are widely used in culinary applications to improve organoleptic properties, including cinnamon (*Cinnamomum zeylanicum* Blume) and laurel (*Laurus nobilis* L.), while others, such as camphor (*Cinnamomum camphora*) and benzoin (*Lindera benzoin* (L.) Blume), are known for their medicinal properties [1].

Many *Lauraceae* species are valued for their essential oils (EOs), which exhibit diverse bioactivities, such as antioxidant, analgesic, anti-inflammatory, antibacterial, antifungal, and insecticidal effects, primarily attributed to the monoterpenes, sesquiterpenes, and aromatic compounds [1,2,3,4,5,6]. The concentration of these phytocompounds varies widely, depending on both intrinsic factors (e.g., plant species and genotype, plant organs, and developmental stage) and extrinsic factors (e.g., geographic origin, climate, soil quality, harvest time, and extraction method) [7,8,9]. This variability significantly influences the biological properties of EOs.

Given the emergence of antibiotic-resistant bacterial strains [10,11], there is a critical need for new natural alternatives to synthetic antibiotics, underscoring the importance of studying these EOs. *Laurus nobilis*, *Litsea cubeba*, and *Cryptocarya* spp. are particularly promising due to their demonstrated antimicrobial activity against both Gram-positive and Gram-negative bacterial strains [1,4,12,13,14,15]. LNEO has shown antimicrobial activity against *Staphylococcus aureus*, *Bacillus* spp., *Enterococcus faecalis*, *Escherichia coli*, *Pseudomonas aeruginosa*, *Salmonella abony*, and *Klebsiella pneumoniae* [16,17,18]. Similarly, LCEO has demonstrated effectiveness against *S. aureus*, *Listeria monocytogenes*, *Enterococcus* spp., *E. coli*, *Cutibacterium acnes*, *Helicobacter pylori*, *Shigella* spp., *Salmonella enterica*, *Salmonella typhimurium*, and *Yersinia enterocolitica* [19,20,21,22,23,24,25]. Conversely, to our knowledge, no studies have been conducted on the antimicrobial activity of CAEOs, even though other species of this genus have been studied [12].

The mechanisms underlying the antibacterial effects of EOs are multifaceted and include disruption of bacterial membranes, increased permeability leading to leakage of intracellular components, inhibition of key metabolic enzymes, interference with nucleic acid synthesis, and protein denaturation [26]. The combined effects compromise bacterial integrity and function, thereby facilitating effective antimicrobial action against various bacterial strains [20,26,27,28].

Generally, leaf and fruit EOs from LN contain 1,8-cineole as the main compound [29]. Instead, EO from the fruits of LC contains high amounts of geranial and neral [22,30,31,32], while different chemotypes have been reported for those from the leaf: 1,8-cineole, linalool, or sabinene chemotypes [33,34]. Four chemotypes were also confirmed for *Cryptocarya agathophylla* EO from leaves: methyl chavicol, methyl eugenol, α-terpinene, and sabinene chemotypes, while a high amount of methyl chavicol characterises the bark [35].

The chemical composition varies not only by plant origin and part, but also across different regions of the world due to factors such as altitude, latitude, annual average relative humidity, and organic carbon content [36,37,38,39]. Regarding LNEO, studies indicate that 1,8-cineole is the dominant compound in most regions, with variable concentrations [16,40,41,42]. For example, in Montecorice (Southern Italy), the oil contains 1,8-cineole (31.9%), sabinene (12.2%), and linalool (10.2%) [16], whereas Greek samples also have high concentrations of 1,8-cineole (30.8%), but also notable amounts of α-terpinyl acetate (14.9%) and α-terpineol (8.0%) [41]. French and Austrian EOs contain high levels of 1,8-cineole, along with bornyl acetate and methyl eugenol [42]. More recent investigations on Moroccan *L. nobilis* essential oil further confirm this chemical variability, describing chemotypes rich in 1,8-cineole alongside significant contributions from sabinene, α-terpinyl acetate, terpinen-4-ol, and other monoterpenes [43,44,45]. These findings highlight the influence of regional and environmental factors on LNEO composition and underline the importance of comparative phytochemical analysis when interpreting biological activity. In contrast, Tunisian LNEO is characterised by high camphor (34.43%) and 1,8-cineole (20.21%) content [42]. Similarly, the EOs from *Lisea cubeba* fruits contain geranial and neral as the main compounds, in varying concentrations, along with other terpenoids. The one from Taiwan contains geranial (24.63–40.38%), neral (19.75–33.57%), and limonene (11.46–31.57%) [46], whereas D-limonene was unexpectedly a lesser constituent in the EO from China (0.7–5.3%) [39]. The EO from the leaves of *C. agathophylla*, an endemic plant of Madagascar, may also have a different chemical composition. For example, the ones from Sahatrona and Morarano forest are rich in methyl chavicol (91.4–94.8%), followed by limonene (0.6–4.2%), while Ampasina forest contains high amounts of methyl eugenol (72.2–80.3%), α-terpinene (3.1–7.8%), methyl chavicol (2.6–5.0%) and limonene (0.7–5.0%) [35].

Identification of the biochemical profile of EOs is essential, as the synergistic action of several phytochemicals within an EO often leads to enhanced or broader antimicrobial efficacy compared to that of individual components [28,47,48]. Thus, the LNEO can inhibit the growth of the microbial strains at lower concentrations than those of its dominant compound, 1,8-cineole [16]. Similarly, the main compound of *Lisea cubeba* EO, citral, is more efficient against *L. monocytogenes* in the presence of lauric acid [49].

By integrating phytochemical analysis with evaluations of antimicrobial efficacy and antibiotic potency, this study aims to elucidate the relationship between EO composition and biological activity. The findings are expected to provide new insights into the potential of *Lauraceae* EOs, both alone and in combination with conventional antibiotics (ampicillin), as effective alternatives for therapeutic or preservative applications, thereby contributing to strategies addressing antibiotic resistance.

In addition to experimental evaluations, this study employs computational methods. The bioinformatic analysis provides guidance in understanding the mechanisms of ligand–receptor (compound–protein) interactions, aiding in the description of experimental validation [50,51]. Furthermore, reports have suggested a potential link between plant phytoconstituents and their antimicrobial properties [52,53]. Therefore, we examined how the main compounds in the EO contribute to inhibiting specific microbial protein targets. Protein target selections for molecular docking analysis are fondly guided by hypothetical and explorative insights into how plant bioactive compounds could exercise their inhibitory potential against the structural biological proteins.

## 2. Results

### 2.1. GC/MS Analysis Results

The results of the chemical characterisation are presented in Table 1. Detailed GC-MS chromatograms and full compound identification tables are provided in the Supplementary Materials (Figures S1–S3 and Tables S1–S3).

Gas chromatography–mass spectrometry (GC/MS) analysis of the three EOs studied revealed that oxygenated monoterpenes predominated in CAEO (60.49%) and LCEO (68.42%). In contrast, over 50% of compounds from LNEO were monoterpene hydrocarbons (56.14%), and a high amount of oxygenated monoterpenes (43.16%) was also observed. CAEO was rich in eucalyptol (52.09%), α-Pinene (10.41%) and limonene (9.09%). For LCEO, in addition to a high concentration of β-Citral (42.98%) and α-Citral (17.81%), limonene was also identified at 19.19%. High amounts of limonene (30.68%) and eucalyptol (28.40%) characterise LNEO.

### 2.2. Antimicrobial Efficacy

**Figure 1:**
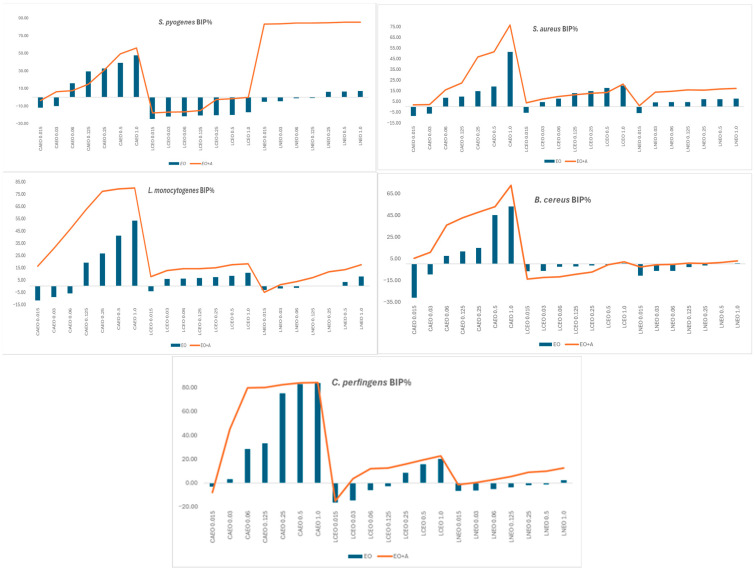
Comparison of Bacterial Inhibition Percentage (BIP%) for CAEO, LCEO, and LNEO applied alone across five Gram-positive strains, single EOs or in combination with antibiotic. BIP% values are shown as qualitative–comparative indicators of antimicrobial activity and are not used for MIC determination or synergy assessment.

**Figure 2:**
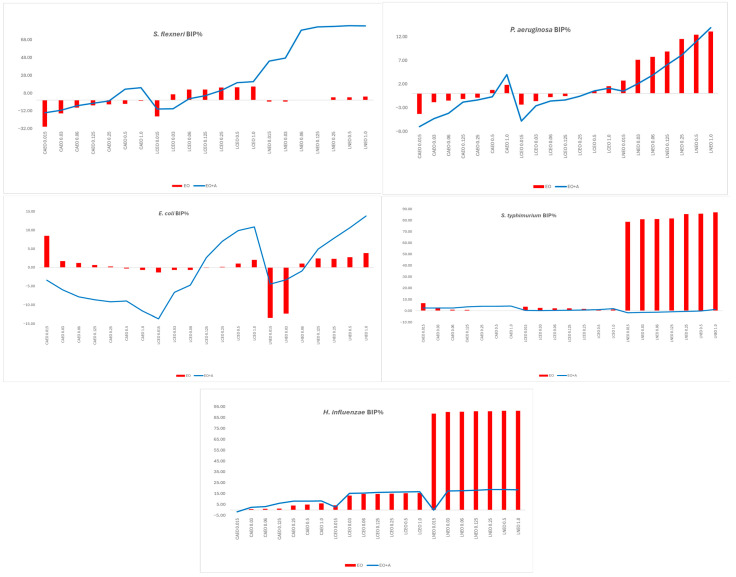
Comparison of Bacterial Inhibition Percentage (BIP%) for CAEO, LCEO, and LNEO applied alone across five Gram-negative strains, single EOs or in combination with antibiotic.

The antimicrobial testing results are illustrated in Figure 1 and Figure 2 as optical density–derived BIP% profiles, which depict relative growth suppression trends across treatments.

Figure 1 and Figure 2 present BIP% profiles to illustrate relative antimicrobial trends of EOs alone and in combination; MIC values and synergy classifications were determined independently and are reported separately.

When tested alone, the three EOs showed apparent differences in antibacterial activity. CAEO was the most active, inducing dose-dependent increases in BIP% across all Gram-positive strains and reaching 50–84% growth inhibition, particularly against *C. perfringens*, *B. cereus*, *L. monocytogenes*, and *S. aureus*. LCEO displayed weak to moderate inhibition, which became evident only at higher concentrations, whereas LNEO exhibited minimal standalone activity against most Gram-positive bacteria. Among Gram-negative species, all oils showed reduced activity, consistent with the protective role of the outer membrane. Notably, LNEO uniquely inhibited *H. influenzae*, with inhibition values ranging from 70% to 91%, while all oils showed weak or negative inhibition against *E. coli*, *S. flexneri*, *S. typhimurium*, and *P. aeruginosa.* Overall, CAEO demonstrated the strongest intrinsic antibacterial activity when tested alone, whereas LCEO and LNEO exhibited limited standalone effects and were therefore further evaluated primarily for their capacity to modulate antibiotic activity in combination assays.

Comparison of bacterial inhibition percentages (BIP%) revealed that, for most strains and concentrations, the combination of EOs with ampicillin resulted in higher growth inhibition compared to the oils tested alone. CAEO showed the strongest intrinsic activity, which was further enhanced in combination assays, reaching inhibition levels of 70–84%. Strains such as *L. monocytogenes*, *B. cereus*, and *C. perfringens* exhibited the most pronounced improvements, with BIP% increases of 20–40% compared to EO alone. Even oils with weak intrinsic inhibition, such as LNEO and LCEO, also displayed increased inhibition when combined with ampicillin, shifting from minimal or negative inhibition values to measurable growth suppression. Overall, the antibiotic improved EO performance across all Gram-positive species, demonstrating additive effects and sub-MIC potentiation not observed in EO-alone treatments.

For Gram-negative strains, combining EOs with ampicillin generally resulted in increased bacterial inhibition compared to EO-alone treatments. *S. flexneri* showed the most significant response, shifting from negative inhibition with EO alone to high inhibition levels (up to 80–88%) in combination experiments, indicating strong potentiation, especially for LCEO and LNEO. *E. coli* and *S. typhimurium* displayed moderate additive effects, with inhibition consistently increasing across all concentrations. *H. influenzae* also showed enhanced growth suppression, particularly with LNEO, while *P. aeruginosa* remained the least responsive, showing only slight improvements due to its inherent resistance. Overall, EO–ampicillin combinations enhanced growth inhibition across Gram-negative species, with the most pronounced potentiation observed for *S. flexneri*.

Across all concentrations and EOs, the addition of sub-MIC levels of ampicillin substantially enhanced antibacterial activity to varying extents, with effects ranging from mild additive effects to pronounced sub-MIC potentiation depending on the EO and bacterial species. Gram-positive bacteria displayed the most consistent enhancement patterns, particularly with CAEO and LNEO. In *S. pyogenes*, *L. monocytogenes*, and *B. cereus*, the presence of ampicillin increased BIP% values by approximately 20–40%, elevating moderate EO-induced inhibition to levels of 60–80%. *C. perfringens* showed the highest absolute inhibition values, reaching 80–84% with CAEO + ampicillin, indicating a substantial enhancement even when the EO alone was already active. *S. aureus* exhibited moderate but reproducible increases in inhibition, with BIP% increasing to 50–75%, consistent with additive effects and sub-MIC potentiation rather than MIC-based synergy.

In Gram-negative bacteria, patterns were strain-dependent and varied in magnitude. *S. flexneri* demonstrated the most pronounced response: EO alone treatments produced negative or minimal inhibition, but EO + ampicillin resulted in 80–88% inhibition, indicating pronounced sub-MIC potentiation of ampicillin activity in a strain-dependent manner, especially with LCEO and LNEO. *E. coli* and *S. typhimurium* showed moderate additive effects, shifting from weak or negative inhibition to consistently positive values (up to 10–13% for *E. coli*, and 8–12% for *S. typhimurium*). Although *H. influenzae* responded moderately to EO alone, the addition of an antibiotic further increased inhibition, maintaining 15–18% activity across all oils. *P. aeruginosa*, known for its multidrug-resistant membrane barrier, showed only mild increases in inhibition (<10%), consistent with its intrinsic resistance mechanisms.

Overall, LNEO and CAEO emerged as the most effective EOs within their respective contexts, with LNEO showing pronounced potentiation effects against Gram-negative strains—particularly *S. flexneri*—and CAEO exhibiting stronger intrinsic and combination-enhanced activity against Gram-positive species such as *L. monocytogenes*, *B. cereus*, and *C. perfringens*. LCEO showed strain-specific potentiation, particularly with *S. flexneri*, but remained weaker in most Gram-positive species. These patterns demonstrate that synergy is not solely determined by EO chemistry but also by bacterial membrane architecture and intrinsic susceptibility. The conversion of weak or negative EO-alone inhibition into elevated BIP% values in the presence of ampicillin highlights the capacity of selected *Lauraceae* EOs to enhance antibiotic-associated growth suppression at sub-MIC levels. For most EO–strain combinations, visible bacterial growth persisted across the tested concentration range, and therefore MIC values were not reached. These findings indicate predominantly sub-inhibitory antimicrobial effects of the oils when applied alone.

MIC determination revealed marked differences among the tested *Lauraceae* EOs. CAEO and LCEO did not achieve complete inhibition of bacterial growth for any of the tested strains within the investigated concentration range, and therefore MIC values were not reached. These oils exhibited predominantly sub-inhibitory, concentration-dependent growth suppression.

In contrast, LNEO demonstrated intrinsic antibacterial activity against selected strains, reaching the MIC threshold (≥90% growth inhibition) for *S. pyogenes*, *S. flexneri*, and *H. influenzae*. In the presence of ampicillin, LNEO showed a pronounced reduction in MIC values for these strains, indicating true MIC-based synergy. For the remaining EO–strain combinations, although enhanced growth suppression was observed in the presence of antibiotic, MIC values were not reached and interactions were therefore classified as potentiation at sub-MIC levels rather than true synergy.

Non-linear concentration–response patterns were observed for several EO–bacteria combinations. Such behaviour is characteristic of multicomponent EOs, where synergistic and antagonistic interactions between constituents, bacterial adaptive responses, and changes in compound bioavailability may occur at higher concentrations. In Gram-negative bacteria, inducible stress and efflux mechanisms can further contribute to non-monotonic inhibition profiles. Therefore, antibacterial responses were interpreted as strain-specific trends rather than uniform dose-dependent effects. Tables S4 and S5 contain OD- and inhibition-based growth suppression profiles of *Lauraceae* EOs applied alone or in combination with ampicillin on Gram-positive and Gram-negative strains. Inhibition values were calculated from optical density measurements and are provided exclusively for qualitative–comparative analysis of antimicrobial trends and sub-MIC potentiation effects. Synergy classification was restricted to combinations exhibiting a true reduction in MIC, whereas OD-based inhibition changes were interpreted as sub-MIC potentiation and are reported separately.

To evaluate the interactions between EOs and ampicillin, a comparative analysis was performed using MIC-based interaction outcomes supported by OD-derived inhibition parameters. Table S6 summarises the minimum inhibitory concentrations (MICs) of each EO alone and in combination with the antibiotic. MIC values were used as the primary criterion to identify true MIC-based interactions, whereas OD-derived percentage enhancement values were included to describe sub-MIC potentiation trends and were not used for synergy classification. This approach allows the identification of strain-dependent interaction patterns, distinguishing combinations that produced clear MIC reductions from those exhibiting only additive effects or limited interaction. The Supplementary Files contain OD- and inhibition-based profiles of *Lauraceae* essential oils applied alone or in combination with ampicillin on Gram-positive and Gram-negative strains. Inhibition values were calculated from optical density measurements and are provided exclusively for qualitative–comparative analysis of antimicrobial trends and sub-MIC potentiation effects. 

Although MIC-based synergy was observed only for selected LNEO–strain combinations, OD-based analyses revealed broader sub-MIC potentiation patterns that were not classified as true synergy. Despite the absence of MICs for most oils when tested alone, several combinations with ampicillin resulted in pronounced MIC reductions and growth suppression, highlighting a clear antibiotic-potentiating effect rather than intrinsic bacteriostatic activity. Only isolated oil–strain combinations reached the MIC threshold, underscoring the strain-dependent nature of EO susceptibility.

The present findings highlight the fundamentally different antimicrobial roles of the investigated *Lauraceae* EOs. CAEO and LCEO primarily acted as modulators of bacterial growth, inducing partial inhibition without achieving complete growth arrest. Such sub-MIC effects are characteristic of complex EO mixtures and do not necessarily translate into bacteriostatic or bactericidal activity when applied alone. Importantly, the absence of MIC values for most EO–strain combinations should not be interpreted as a lack of biological relevance. On the contrary, sub-inhibitory interactions are increasingly recognised as critical contributors to antibiotic potentiation, as they may alter membrane permeability, metabolic activity, or stress response pathways, thereby sensitising bacteria to conventional antibiotics.

**Figure 3:**
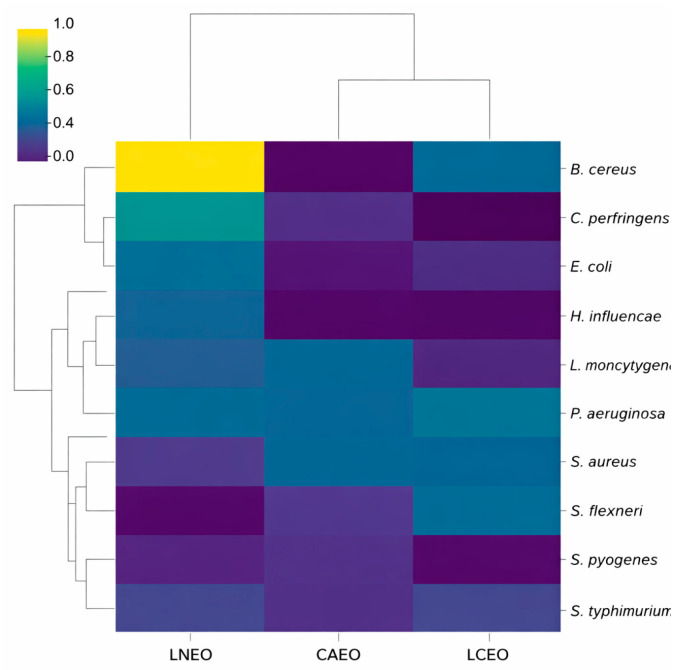
A hierarchical clustered heatmap showing the similarity relationships between the three EOs (CAEO, LCEO, LNEO) and the tested bacterial strains based on a combined interaction profile integrating MIC-based interaction outcomes and normalised sub-MIC potentiation parameters. Rows (bacterial strains) and columns (EOs) are clustered using Euclidean distance and complete linkage. LNEO forms a distinct branch, reflecting a unique interaction profile characterised by pronounced sub-MIC potentiation and selected MIC-based interactions against specific strains, including *P. aeruginosa*, *S. pyogenes*, and *H. influenzae*. CAEO and LCEO cluster together, indicating comparable and generally moderate interaction profiles. Bacterial strains also segregated into distinct response clusters, reflecting differential sensitivity to EO–ampicillin combinations rather than uniform synergy levels.

In this context, LNEO emerged as the only oil exhibiting intrinsic antibacterial activity against selected strains, enabling clear MIC-based synergy with ampicillin. For CAEO and LCEO, the observed enhancement of antibiotic-induced growth suppression reflects potentiation effects rather than true synergy, underscoring the importance of distinguishing between MIC-based interactions and sub-MIC modulation when evaluating EO–antibiotic combinations. Cluster analysis of the combined interaction matrix revealed clear groupings of EOs based on their overall antibacterial response profiles (Figure 3). LNEO formed a distinct, isolated cluster, reflecting its pronounced interaction patterns in combination with ampicillin across multiple bacterial strains. These patterns were driven primarily by strong sub-MIC potentiation effects and selected MIC-based interactions rather than uniform broad-spectrum synergy. In contrast, CAEO and LCEO clustered closely, indicating comparable, generally moderate interaction profiles characterised mainly by additive effects and sub-MIC potentiation across most strains.

### 2.3. Molecular Docking Analysis

The docking results presented in Table 2 correspond to the lowest binding energy, which reflects the binding affinity of each ligand to the respective microbial protein targets. The analysis showed amino acid residues that interact mainly in hydrophobic bonds (π-sigma, alkyl, π-alkyl, van der Waals) and hydrophilic bonds (hydrogen, carbon-hydrogen) with the ligands. The lowest binding energy for each of the protein targets ranges from −4.3 to −5.8 kcal/mol (2WLA), −4.7 to −7.1 kcal/mol (3U2D), −4.4 to −6.2 kcal/mol (3UUE), −5.8 to −8.0 kcal/mol (5TZ1), −5.8 to −8.0 kcal/mol (6V7X), −4.8 to −6.3 kcal/mol (7EIP), respectively.

**Figure 4:**
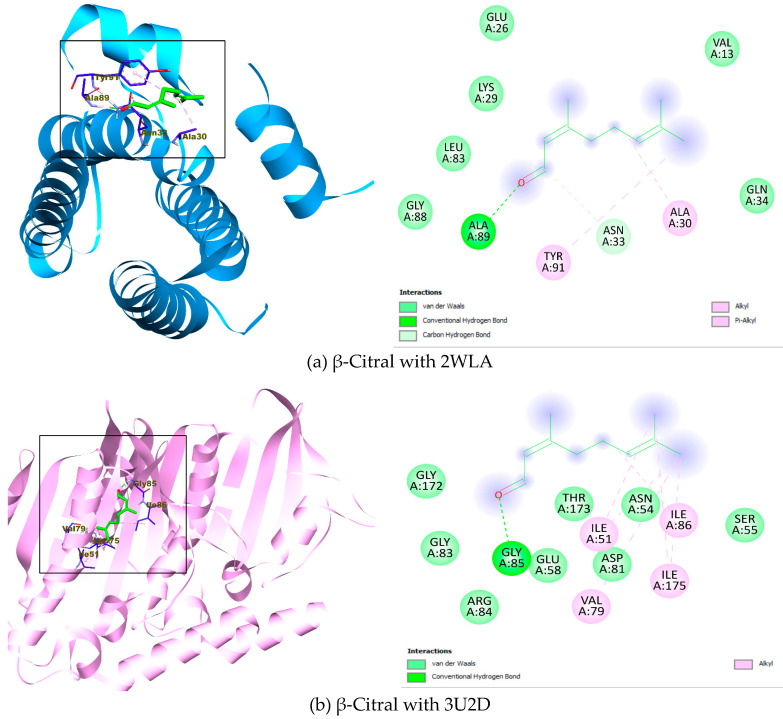

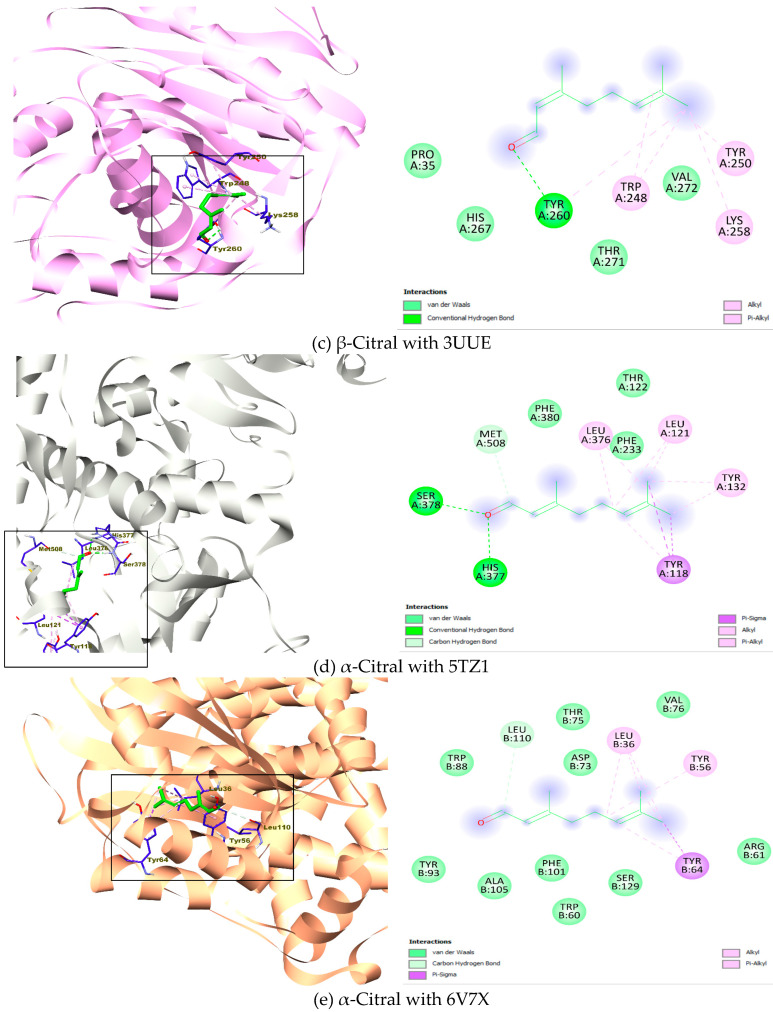

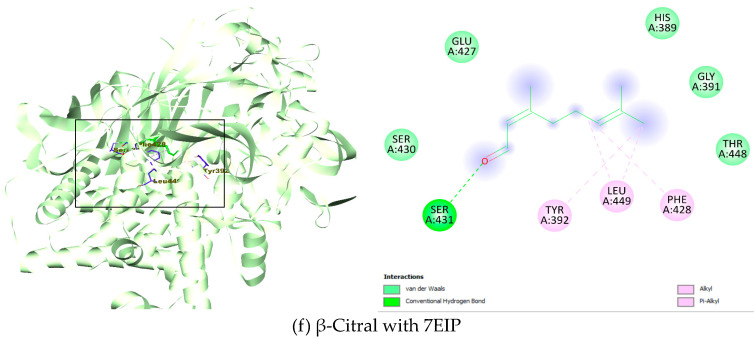
3D and 2D representation of the ligand–protein complex for the best binding pose.

The depicted binding interactions for the best ligand poses against each protein target are shown in Figure 4. The insight from the interactions of the ligands to the respective protein targets is favoured in both hydrophobic bonds and hydrophilic bonds, wherein β-Citral with 2WLA (Ala A: 30, Asn A: 33, Ala A: 89, Tyr A: 91), β-Citral with 3U2D (Ile A: 51, Val A: 79, Gly A: 85, Ile A: 86, Ile A: 175), β-Citral with 3UUE (Trp A: 248, Tyr A: 250, Lys A: 258, Tyr A: 260), α-Citral with 5TZ1 (Tyr A: 118, Leu A: 121, Tyr A: 132, Leu A: 376, His A: 377. Ser A: 378), α-Citral with 6V7X (Leu B: 36, Tyr B: 56, Tyr B: 64, Leu B: 110) and β-Citral with 7EIP (Tyr A: 392, Phe A: 428, Ser A: 431, Leu A: 449) (Figure 4). Cumulatively, the docking analysis underscores the efficient binding of the high abundant compounds in the three EOs with bacterial protein targets.

## 3. Discussion

The chemical composition of *Lauraceae* EOs varied widely, depending on numerous factors related to plant characteristics, environmental conditions, and production type.

The present study focused on three commercial EOs from the *Lauraceae* family (CAEO, LCEO, LNEO), demonstrating that they contain high levels of monoterpenes, which are significant components due to their biological properties, such as antimicrobial, antifungal, antiviral, anticancer, and anti-inflammatory [55,56,57]. The predominance of oxygenated monoterpenes was observed for CAEO and LCEO, while LNEO was rich in hydrocarbon monoterpenes. Although CAEO and LCEO shared the same major chemical class, their compositional profiles differed, reflecting distinct botanical origins. Compared with the literature, similarities and differences in biochemical profiles of the EOs studied are observed. Regarding CAEO, the data remains insufficient. Although four chemotypes (methyl chavicol, methyl eugenol, α-terpinene, and sabinene) were initially identified [35], another chemotype, limonene, was subsequently discovered [58]. In contrast, the current study highlighted a high eucalyptol level and a limonene percentage of only 9.09%. The elevated levels of eucalyptol observed may reflect a defence response in young leaves [59,60,61].

Research has consistently highlighted the significant role of 1,8-cineole in LNEO, which is often present at high concentrations, making it a key component of the oil’s therapeutic potential. Studies indicate that LNEO from Greek, Georgian, and Italian sources contains approximately 30% 1,8-cineole [16,40,41], while those from Montenegro, France, and Austria show even higher levels, around 40% [29,42]. Other compounds include sabinene, linalool, α-terpineol, terpinen-4-ol, α-pinene, and β-pinene, at varying concentrations across different regions of the world [16,29,40,41]. The chemical profile of LNEO observed in the present study is consistent with previously reported Mediterranean chemotypes, in which 1,8-cineole represents a major constituent. Comparative studies from Southern Europe and North Africa, including well-documented Moroccan samples, demonstrate that while 1,8-cineole frequently dominates LNEO composition, its concentration and the relative abundance of accompanying monoterpenes such as sabinene, linalool, α-terpineol, terpinen-4-ol, α-pinene, and β-pinene vary markedly among regions [43,44,45].

Conversely, the commercial LNEO analysed from Romania showed a composition rich in limonene (30.68%) and 1,8-cineole (28.40%), along with moderate amounts of camphor (9.09%), β-myrcene (6.01%), and ocimene (5.73%). Recognising these differences is essential for selecting the most effective oils for specific health purposes.

The biochemical profile of LCEO from fruits is rich in β-Citral, α-Citral, and limonene, with varying concentrations observed across different regions, as documented in the literature [22,31,32,39,46]. Specifically, the LCEO from China contains up to 78.7% α-Citral and 87.4% β-Citral [39], whereas those from Taiwan are approximately half as high [46]. However, the one from Taiwan has a higher limonene concentration (11.46–31.57%) than the one from China [39]. The findings of the current study closely resemble those of Taiwan LCEO, indicating a similar plant origin for the commercial oil analysed. Understanding compositional differences is crucial for developing region-specific applications in therapeutic contexts. Citral is known for its antimicrobial, antioxidant, anticancer, antidiabetic, and anti-inflammatory properties [62,63]. Limonene also demonstrates a broad spectrum of benefits, such as antioxidant, antinociceptive, anticancer, antidiabetic, antiviral, and gastroprotective effects [64]. The synergistic action of citral and limonene reinforces the recommendation of LCEO for antimicrobial applications, enhancing its therapeutic efficacy [65].

EOs exhibit significant antimicrobial activity. Key compounds such as citral, limonene, 1,8-cineol, and camphor have been studied for their antibacterial and antifungal properties, demonstrating varying degrees of effectiveness against a range of microorganisms [59,66,67,68,69,70,71]. Although these EOs exhibit antimicrobial activity against a broad spectrum of bacteria, the effective values varied widely, often reflecting the influence of both bacterial cell wall structure and EO composition. Typically, Gram-negative bacteria tend to be more resistant than Gram-positive bacteria to EOs [16,17,18] due to the presence of an outer membrane that restricts the penetration of hydrophobic compounds [72]. For example, LNEO has shown MIC values of 0.2–0.4 µL/mL against *B. cereus* and *S. aureus*, and 0.4–0.8 µL/mL against *E. coli* and *P. aeruginosa* [16]. Another study demonstrated that concentrations of 250–500 μg/mL inhibited the growth of Gram-positive bacteria such as *S. aureus*, *B. subtilis*, and *E. faecalis*, whereas higher concentrations (1000 μg/mL) were required against Gram-negative species such as *K. pneumoniae*, *S. abony*, and *P. aeruginosa* [18]. Instead, the literature on MIC values of LCEO is inconsistent. For instance, Chen et al., [73] showed that MIC values were 0.78 μL/mL and 6.25 μL/mL for *B. subtilis* and *S. aureus*, with higher values observed against Gram-negative strains, specifically ranging from 6.25 to 200 μL/mL, indicating *Pseudomonas aeruginosa* as the most resistant strain. In contrast, Wang et al. [26] found similar MIC values for both Gram-positive and Gram-negative bacteria (0.8 mg/mL), and even lower values for the Gram-negative strain *S. typhimurium* (0.4 mg/mL).

However, determining MICs for a specific type of EO against a target bacterial strain is challenging due to significant variation in the oil’s chemical profile [16,40,41,42,74].

As complex mixtures of terpenoids, EOs frequently display strain-dependent MIC values, and their antimicrobial efficacy can differ markedly even against closely related bacterial species. Gram-positive bacteria are generally more susceptible to EO compounds. However, some studies suggest that LCEO and LNEO may be more effective against Gram-negative bacteria [23,26]. For example, Wang et al. [26] sustained MIC values of LCEO of 0.8 mg/mL against *L. monocytogenes* and *B. cereus*, and of 0.4 mg/mL against *S. typhimurium*. Similarly, Mei et al. [23] reported a larger inhibition zone against *E. coli* O157:H7 and *S. enterica* than against Gram-positive bacteria, with MIC values of 0.9 μg/mL against both bacteria. Regarding LNEO, Mkaddem Guedri et al. [42] demonstrated that Tunisian EO had a lower MIC against *K. pneumonial*, *E. coli* and *S. enterica* (0.004 mg/mL) than the Gram-positive bacteria, specifically *B. subtilis*, *S. aureus* and *L. monocytogenes* (0.01 mg/mL) [42]. The findings of the present study are broadly consistent with these reports [23,26,42], highlighting pronounced strain-dependent differences rather than a strict Gram-type selectivity. In our assays, both LNEO and LCEO exhibited low MIC values against selected Gram-negative strains, including *P. aeruginosa*, *H. influenzae*, and *S. typhimurium* (0.015–0.03 µL/mL), while higher concentrations were required for *B. cereus* (1.00 µL/mL). In contrast, CAEO displayed stronger intrinsic activity against Gram-positive bacteria, with MIC values ranging from 0.03 to 0.5 µL/mL. In contrast, activity against Gram-negative strains was generally lower (0.015–0.25 µL/mL), with *S. flexneri* remaining resistant under the tested conditions.

However, this cannot be considered a rule, since each bacterial strain responds differently to the chemical compounds in EOs. Studied LNEO needs a higher concentration against *C. perfringens* at 1.00 µL/mL and a moderate one against *S. pyogenes* at 0.25 µL/mL, while *L. monocytogenes* was very susceptible to the chemical composition of this EO (MIC = 0.015 µL/mL), even though all are Gram-positive bacteria. On the other hand, the MIC value against *S. flexneri* was 0.5 µL/mL. Similarly, Da Silviera et al. [75] found MIC values of LNEO against *S. aureus* of 5.00–10.00 g/L and against *L. monocytogenes* of only 1.25–2.5 g/L, whereas against *P. aeruginosa*, it was 10.00 g/L. Caputo et al. [16] demonstrated that *B. cereus* is more susceptible than *S. aureus* to LNEO, while *E. coli* requires a higher concentration than Gram-positive bacteria, at 0.8 µL/mL. For the investigated LCEO, pronounced variability in antibacterial responses was observed within the same bacterial groups, highlighting strain-dependent sensitivity rather than uniform susceptibility patterns. Gram-positive bacteria generally exhibited lower responsiveness to LCEO, with measurable growth inhibition detected only at higher concentrations, while *S. pyogenes* showed no appreciable response under the tested conditions. In contrast, among Gram-negative bacteria, *S. typhimurium* and *P. aeruginosa* showed greater sensitivity to LCEO, exhibiting detectable growth suppression at lower concentrations, whereas E. coli was the least responsive, requiring substantially higher concentrations to achieve comparable inhibition levels. CAEO similarly demonstrated strain-dependent antibacterial effects. Noticeable growth suppression was observed against *S. typhimurium* and *H. influenzae*, while among Gram-positive bacteria, *C. perfringens* exhibited the highest susceptibility. Conversely, *L. monocytogenes* showed the lowest responsiveness, requiring the highest tested concentrations to achieve measurable inhibition.

The variability in antibacterial responses emphasises the need for a tailored approach when using EOs for therapeutic applications, as different bacterial strains respond uniquely to specific compounds. Understanding how particular compounds in EOs interact with various bacterial strains at the molecular level can inform the development of more effective therapeutic applications. Additionally, investigating the additive or potentiating effects of minor compounds may lead to the development of more potent formulations.

In addition to exploring interactions among natural compounds, attention has increasingly focused on the capacity of EOs to modulate the antibacterial activity of conventional antibiotics. The literature indicates that both LNEO and LCEOs exhibit either synergistic or additive actions in their interactions with antibiotics. Nafis et al. [76] reported the synergistic activity of LNEO with ciprofloxacin and vancomycin against *S. aureus*, *B. subtilis*, *E. coli*, *P. aeruginosa*, and *K. pneumoniae*. Similarly, Sena et al. [77] demonstrated that LNEO exhibited synergistic activity with ceftriaxone and additive effects with ampicillin and ciprofloxacin against *E. coli*. For *P. aeruginosa*, synergistic activity was noted with ciprofloxacin and additive action with ampicillin [77]. In contrast to these reports, the present study did not observe enhanced growth inhibition for *P. aeruginosa* when LNEO was combined with ampicillin, highlighting the importance of antibiotic class and experimental context in determining interaction outcomes. Instead, pronounced strain-dependent potentiation effects were observed for selected Gram-positive and fastidious bacteria, including *S. pyogenes*, *H. influenzae*, and *L. monocytogenes*, while more modest additive responses were detected for *S. aureus* and *E. coli*. For LCEO, interaction outcomes were likewise strain-dependent. Enhanced growth suppression was observed for *S. flexneri* and *C. perfringens* in the presence of ampicillin, whereas more limited additive effects were detected for *S. aureus* and *P. aeruginosa*. Comparable variability has been reported for other antibiotic classes, such as tetracycline and oxytetracycline hydrochloride, in combination with *Lauraceae* EOs [78]. Overall, these findings indicate that EO–antibiotic interactions are highly context-dependent and are influenced by both the oil’s chemical composition and the bacterial target. Rather than universal synergy, the data support a model of selective potentiation and additive effects, which may inform future studies aimed at optimising EO–antibiotic combinations under defined conditions.

The cluster analysis and composite interaction profiling provide a comprehensive overview of the differential potentiation capacity of the tested EOs. The composite score was used as an exploratory clustering tool to visualise relative interaction patterns rather than as a definitive measure of antimicrobial synergy.

LNEO demonstrated a distinct and pronounced interaction profile, forming a separate cluster in the hierarchical analysis and showing elevated composite interaction values. This behaviour is consistent with its chemically diverse terpene profile, which may influence bacterial growth responses and antibiotic-associated inhibition. CAEO and LCEO, although chemically distinct, displayed more similar interaction profiles and clustered together, indicating comparable and generally moderate levels of interaction with ampicillin.

The molecular docking results from this study indicate that the lowest-scoring binding energies are predicted to have high inhibitory potential against the protein targets. The selected targets for docking play central roles in exercising the pathogenicity of the microorganism (Bacteria and fungi). Dpr (DNA-binding protein from starved cells-like peroxide resistance protein) plays a critical role in bacterial protection against oxidative stress, particularly in catalase-deficient pathogens such as *Streptococcus pyogenes*. Dpr, a member of the Dps protein family, protects bacteria by binding and oxidising Fe^2+^, preventing the formation of hydroxyl radicals that damage DNA. It forms a ferritin-like dodecamer with a central iron-storage cavity and contains ferroxidase centres at subunit interfaces that catalyse iron oxidation. Studies show that loss of Dpr increases bacterial vulnerability to oxidative stress, a crucial factor during immune responses. Therefore, disrupting the Dpr structure or its iron-binding capacity may enhance antibacterial effects by making bacteria more susceptible to oxidative damage and antibiotics. [79]. DNA gyrase is an essential type II topoisomerase vital for bacterial DNA replication, transcription, and chromosome segregation, introducing negative supercoils in an ATP-dependent process. It has two subunits, GyrA and GyrB, necessary for maintaining DNA topology. Inhibiting gyrase disrupts supercoiling, halts replication, impairs transcription, and causes bacterial death. Its absence in higher eukaryotes makes it a validated antibacterial target, as exemplified by antibiotics such as fluoroquinolones and aminocoumarins. Blocking the GyrB ATP-binding pocket inhibits enzyme activity and bacterial growth, highlighting its importance in antibacterial drug discovery [80]. Mono- and diacylglycerol lipases are enzymes that regulate lipid metabolism and membrane processes. They have a “lid” domain covering the active site that changes shape upon interacting with lipid–water interfaces, exposing the catalytic cleft for substrate binding and hydrolysis. This mechanism stabilises the enzyme in water and allows it to interact with hydrophobic substrates. Disrupting the lid or active site could impair lipid processing and membrane function, making these enzymes targets for antimicrobial compounds derived from EOs, as demonstrated in fungal strains [81]. DNA gyrase is an essential bacterial type II topoisomerase that plays a central role in DNA replication, transcription, and chromosome segregation by introducing negative supercoils into DNA. This enzyme is indispensable for maintaining DNA topology during rapid bacterial growth and cell division, making it a well-validated antibacterial target. Inhibition of DNA gyrase disrupts DNA supercoiling homeostasis, leading to impaired replication fork progression and ultimately bacterial cell death. Consequently, DNA gyrase has been extensively targeted by several clinically important antibacterial agents, especially *Candida* strains. In this context, the observed binding of bioactive constituents to the DNA gyrase active site supports a plausible molecular mechanism underlying the antibacterial effects detected *in vitro*, particularly against strains exhibiting pronounced growth inhibition [82]. Quorum sensing regulates bacterial behaviours like virulence, biofilm formation, and anti-phage defences. Recent evidence shows bacteriophages counter these pathways. In *Pseudomonas aeruginosa*, the phage-encoded anti-activator protein Aqs1 inhibits LasR, a key quorum-sensing regulator, thereby silencing multiple defence mechanisms. Aqs1 binds LasR’s DNA-binding domain, blocking its interaction with target promoters and reducing gene expression. This allows phages to evade defences and suggests quorum-sensing regulators as targets for antimicrobial strategies to modulate bacterial pathogenicity and resilience without killing bacteria [83]. Chondroitinase ABC I (ChABC I) is a polysaccharide lyase that degrades chondroitin sulphate and related glycosaminoglycans via β-elimination, cleaving the β-1,4-glycosidic bond between D-glucuronic acid and N-acetyl-galactosamine units. This produces unsaturated disaccharides and is crucial for remodelling extracellular polysaccharides. Structural studies show ChABC I has a catalytic cleft with conserved residues (Arg500, His501, Tyr508, His561, Arg560) that recognise substrates. It prefers chondroitin sulphate A and C, recognising sulfation patterns. Since degradation of chondroitin sulphate affects bacterial interactions and host tissues, ChABC I is a key target to assess how EO constituents might inhibit enzymes involved in bacterial persistence and pathogenicity, with docking providing mechanistic insights [84].

The docking study of the ligands with the bacterial protein targets (PDB ID: 2WLA, 3U2D, 3UUE, 5TZ1, 6V7X, 7EIP). The core mode of interaction predicted from the docking outcomes of the ligand-protein complexes was explored in terms of hydrophobic and hydrophilic bonds. β-Citral is predictably binding to the amino acid residues of the DNA gyrase protein binding pocket (Gly A: 85, amongst other binding types). Again, the strong hydrogen bond between α-Citral and the imidazole ring of His A: 377 is an indication of potency by the bioactive compound towards inhibiting the CYP51 activity in addition to other interactions (Figure 4d). Overall, the plausible binding positions of these bonds were mainly stabilised by van der Waals, alkyl, pi-alkyl, pi-sigma, hydrogen, and carbon-hydrogen bonds (Figure 4), all of which interacted with key functional groups of the compounds and the amino acid residue (Table 2). In toto, the collective contributions of the bioactive compounds identified in *Lauraceae* EO could be suggested to have antimicrobial activity and be corroborated by the *in vitro* assay.

Despite the promising antibacterial activity observed with *Lauraceae* EOs, their direct pharmaceutical application is challenged by chemical instability, volatility, limited solubility, and variable bioavailability, which can limit their clinical relevance. Recent advances in delivery technologies, such as chitosan-based encapsulation systems, have been highlighted as effective strategies to enhance the stability, controlled release, and bioavailability of EOs, thereby potentially improving their translational applicability in antimicrobial formulations [85]. A limitation is reliance on *in vitro* assays, which may not fully reflect the stability, bioavailability, or activity of EOs under physiological conditions. Future research should evaluate delivery strategies and *in vivo* models to assess the translational relevance of EO–antibiotic combinations. These findings serve as proof-of-concept; further studies on cytotoxicity, pharmacokinetics, and delivery strategies are necessary before application.

From a translational perspective, the findings of the present study should be interpreted with caution. The interaction and potentiation effects observed were obtained under *in vitro* conditions, and the effective EO concentrations may not be readily achievable or sustainable *in vivo*. Moreover, cytotoxicity and selectivity toward host cells were not assessed in this study. Consequently, the results are intended to provide mechanistic and comparative insight into strain-dependent EO–antibiotic interactions rather than direct therapeutic recommendations. Future studies should address biocompatibility, optimised delivery systems, and *in vivo* validation to better evaluate translational potential.

## 4. Materials and Methods

### 4.1. Chemicals

The chemicals used were purchased from Sigma-Aldrich Chemie GmbH, Munich, Germany. All reagents were analytically pure. All culture media used for microbiological analyses were purchased from Oxoid Limited (Thermo Fisher Scientific Inc., Waltham, MA, USA).

### 4.2. Determination of Chemical Profile by GC-MS Analysis

EO (EO) samples were chemically characterised using Gas Chromatography-Mass Spectrometry (GC-MS) on a Shimadzu QP 2010 Plus instrument (Shimadzu Corporation Columbia, SC, USA) equipped with a low-polarity AT-5MS capillary column (5% phenyl–95% dimethylpolysiloxane) (30 m, 0.32 mm, 1 µm). The analysis followed established methodologies from previous studies [86]. Helium served as the carrier gas at a flow rate of 1 mL/min, with the injector temperature set at 250 °C and the ion source temperature at 220 °C. A temperature gradient was utilised for compound separation, beginning with an initial oven temperature of 40 °C (held for 1 min), then increasing to 210 °C at a rate of 5 °C/min, followed by a 5 min hold. The injection volume was 1 μL of a 2% EO/hexane solution, using a 1:50 split ratio.

Identification of the separated compounds was performed by mass spectrometry in MS scan mode (35–500 *m*/*z*) using the NIST database. At the same time, their quantification was performed using the normalised area method in SIM mode. A concordance of at least 90% was observed between the detected compounds and the database. The linear retention index (LRI) was calculated using the normal alkane RI for the same type of column. The relative composition of the EOs was determined from normalised peak areas. For each EO, the sum of all detected peak areas was considered as 100%, and the relative abundance of each compound was expressed as a percentage of this total. Retention times (R.T.) are reported in minutes and correspond to the elution time of each compound under the applied chromatographic conditions [54].

### 4.3. Antimicrobial Activity

The antimicrobial activity of EOs used in this study was evaluated through broth microdilution against Gram-positive and Gram-negative bacterial strains. The Gram-positive ATCC strains tested were *S. pyogenes* (ATCC 19615), *S. aureus* (ATCC 25923), *C. perfringens* (ATCC 13124), *L. monocytogenes* (ATCC 19114), and *B. cereus* (ATCC 10876). The Gram-negative strains taken into the study were *P. aeruginosa* (ATCC 27853), *S. flexneri* (ATCC 12022), *E. coli* (ATCC 25922), *S. typhimurium* (ATCC 14028), and *H. influenzae type B* (ATCC 10211).

A fresh culture was created in Brain Heart Infusion (BHI) broth (Oxoid, CM1135) to assess the antimicrobial effects of EOs, as outlined in our previous research [87]. An overnight bacterial culture was initially diluted (10^−3^) to obtain a working suspension, which was subsequently adjusted to a 0.5 McFarland standard (approximately 1.5 × 10^8^ CFU/mL). This standardised suspension was used directly for the broth microdilution assay. EOs were dissolved in dimethyl sulfoxide (DMSO) to ensure homogeneous dispersion in aqueous culture media. The final DMSO concentration did not exceed levels shown to affect bacterial growth, and solvent controls were included in all experiments. The EOs were directly added to the bacterial suspension at concentrations of 0.015 mg/mL, 0.03 mg/mL, 0.06 mg/mL, 0.125 mg/mL, 0.25 mg/mL, 0.5 mg/mL, and 1.0 mg/mL. These concentrations were selected based on earlier studies and a literature review, aiming to cover a diverse range to identify potential minimum inhibitory concentrations (MICs). All assays were conducted in biological triplicate, and appropriate solvent controls were used to account for DMSO and potential oil-induced turbidity. Optical density (OD) measurements were used as indicators of relative bacterial growth suppression and were not the sole criterion for minimum inhibitory concentration (MIC) determination.

MIC values were defined as the lowest concentration inhibiting visible bacterial growth after incubation. The MIC, which is the lowest concentration of the compound that inhibits visible microbial growth, was determined by measuring optical density (OD) through a spectrophotometric method [88]. For result interpretation, two metrics were calculated: bacterial growth percentage (BGP) and bacterial inhibition percentage (BIP), using Formulas (1) and (2), respectively.

(1) $$\mathrm{BGP}\;(\%)=\frac{OD\;sample}{OD\;negative\;control\;sample}\times 100$$

(2) BIP (%)=100−BGP (%)

where OD sample represents the optical density at 540 nm as a mean value of triplicate readings; OD negative control represents the optical density at 540 nm as a mean value of triplicate readings for the selected bacteria in BHI.

BIP% values were calculated to provide a qualitative–comparative visualisation of antimicrobial effects across concentrations and treatments and were not used to define MIC or classify synergistic interactions.

### 4.4. Evaluation of Ampicillin Potentiation by EOs Using MIC-Based Interaction Analysis and Sub-MIC Growth Inhibition

Ampicillin was selected as a representative β-lactam antibiotic to evaluate whether *Lauraceae* EOs are capable of modulating antibiotic-associated growth inhibition under conditions of reduced baseline susceptibility. Although β-lactam antibiotics generally exhibit limited activity against several Gram-negative bacteria due to the presence of an outer membrane and intrinsic resistance mechanisms, their use in the present study was intentional. This approach allowed the assessment of EO-associated potentiation and interaction patterns under stringent conditions rather than optimal antibiotic susceptibility. The use of ampicillin at sub-inhibitory concentrations enabled the investigation of strain-dependent responses to EO–antibiotic combinations without implying universal efficacy of β-lactams against Gram-negative pathogens. Accordingly, observed interaction effects are interpreted as context-specific and exploratory, highlighting differential bacterial responsiveness rather than broad-spectrum antibiotic enhancement.

The interaction between EOs and ampicillin was tested using the checkerboard method, as previously described in the literature [87,89]. Bacterial inocula were prepared from overnight cultures and adjusted to a 0.5 McFarland standard (approximately 1.5 × 10^8^ CFU/mL). This standardised suspension served as the stock inoculum. It was further diluted in Mueller–Hinton broth during plate setup to achieve a final bacterial concentration of approximately 1 × 10^5^ CFU/mL per well, in accordance with CLSI recommendations. [90]. EOs (CAEO, LCEO, and LNEO) were added at final concentrations ranging from 0.015 to 1.0 mg/mL, while ampicillin (Sigma-Aldrich, Merck KGaA, Darmstadt, Germany) was applied at sub-MIC levels predetermined for each ATCC strain. Plates were incubated at 37 °C for 24 h under aerobic conditions, and all assays were performed in triplicate [90].

In all interaction experiments, ampicillin was deliberately used at concentrations below the MIC established for each ATCC strain. These sub-MIC antibiotic levels were combined with EO-containing DMSO solutions to ensure that any observed changes in bacterial growth were attributable to EO–antibiotic interactions rather than to the antibiotic alone. Plates were incubated for 24 h at 37 °C under aerobic conditions, and all experiments were performed in triplicate.

After incubation, bacterial growth was assessed by measuring optical density (OD) spectrophotometrically. MIC values were defined as the lowest concentrations that prevented visible bacterial growth (≥90% growth inhibition). MIC-based interactions were considered only when a clear reduction in MIC was observed in the presence of ampicillin.

For EO–strain combinations in which MIC values were not reached, interaction effects were evaluated using OD-derived inhibition parameters. Percentage enhancement in growth inhibition was calculated according to Equation (1) and was used exclusively to describe potentiation effects at sub-MIC levels. These OD-based parameters were not used to define MIC values or to classify synergistic interactions.

BIP% values were calculated to provide a qualitative–comparative visualisation of antimicrobial activity trends across concentrations and treatments and were not employed for MIC determination or synergy classification and the enhamncement is calculated as Formula (3).

(3) $$\mathrm{Enhancement}\;(\%)=\frac{OD\;antibiotic-OD\;combination}{OD\;antibiotic}\times 100$$

Fold-change analysis was employed as a descriptive indicator to compare EO concentrations required to achieve comparable growth inhibition in the absence and presence of sub-inhibitory ampicillin. Similar fold-change–based approaches have been previously used to describe interaction trends and relative potentiation effects between natural products and antibiotics, without implying definitive pharmacological synergy [91,92]. Given the complex and multicomponent nature of EOs, fold reduction values were interpreted cautiously and used exclusively as comparative descriptors rather than as validated synergy metrics.

### 4.5. Molecular Docking

Molecular docking was conducted using PyRx—Python Prescription 0.8. The chemical structure of the fourteen (14) high abundant compounds in the EOs were retrieved from https://pubchem.ncbi.nlm.nih.gov/ [93] in SDF format, accessed on 18 May 2025 and docked against six (6) protein targets. The 3D crystal structure of the proteins (PDB ID: 2WLA [Dpr protein], 3U2D [DNA gyrase], 3UUE [mono- and diacylglycerol lipase], 5TZ1 [CYP51], 6V7X quorum-sensing anti-activator protein], 7EIP [Chondroitinase ABC I]) were obtained from the Protein Data Bank, https://www.rcsb.org/ [94] in legacy PDB format, accessed on 24 August, 2025. The protein targets were the crystallised structures of *Streptococcus pyogenes*, *Staphylococcus aureus*, *Malassezia globosa*, *Candida albicans*, *Pseudomonas aeruginosa* and *Proteus vulgaris*. Each protein was prepared for docking by removing solvent, ions, co-crystallised ligands, and any heteroatoms in UCSF Chimaera (version 1.17.3), then saved as a .pdb file.

The compounds were imported into the PyRx software (PyRx software (Python Prescription 0.8) Autodock Ligand (.pdbqt) and all selected to be minimised with the addition of energy of minimisation (universal force field) before converting all the compounds to AutoDock Ligand (.pdbqt) (PyRx software (Python Prescription 0.8) Autodock Ligand). Next, the proteins were each loaded and prepared as macromolecules for AutoDock, then converted to .pdbqt. The virtual screening of the compounds against each protein is completed using the Vina Wizard, which starts when the grid box is maximised. The grid coordinates for each protein was resolved cover the protein targets, 2WLA (center_x = 59.9274, _y = 110.5668, _z = 138.4204; size_x = 35.6550, _y = 35.2279, _z = 53.9062), 3U2D (center_x = 16.4140, _y = 0.5366, _z = 11.5998; size_x = 67.3296, _y = 71.1591, _z = 66.9449), 3UUE (center_x = −2.6334, _y = 0.1542, _z = 13.4958; size_x = 44.9844, _y = 51.3351, _z = 47.7857), 5TZ1 (center_x = 60.2572, _y = 50.5836, _z = 21.7248; size_x = 68.3750, _y = 87.1145, _z = 104.8505), 6V7X (center_x = 20.6172, _y = 9.5625, _z = −20.5775; size_x = 51.3252, _y = 60.0891, _z = 71.2674), 7EIP (center_x = −8.6021, _y = 25.3648, _z = −32.0769; size_x = 80.6849, _y = 112.8706, _z = 86.7821). The exhaustiveness was 8. BIOVIA Discovery Studio Visualizer v21.1.0.20298 (BIOVIA, San Diego, CA, USA) was used to visualise the binding interaction and poses (2D and 3D) of the docking, respectively.

### 4.6. Statistical Analysis

All experiments were performed in biological triplicate, and results are expressed as mean values ± standard deviation (SD). Optical density (OD) measurements and inhibition-related parameters were statistically analysed to evaluate differences between treatments and controls. One-way analysis of variance (ANOVA) was applied to assess statistically significant differences among multiple groups, followed by Tukey’s honestly significant difference (HSD) post hoc test for pairwise comparisons when appropriate using JASP 0.19.3.0.

## 5. Conclusions

In summary, this study provides a multi-method evaluation of Lauraceae EOs as modulators of antibiotic-associated growth inhibition across diverse bacterial strains. By integrating chemical profiling, *in vitro* interaction assays, and exploratory clustering analyses, the work highlights pronounced strain-dependent response patterns while avoiding overgeneralisation or premature translational claims. These findings provide a framework for future mechanistic and formulation-focused studies to refine EO–antibiotic combinations under defined experimental conditions. The three *Lauraceae* EOs displayed distinct chemical and biological profiles, with LNEO consistently providing the strongest synergistic enhancement of ampicillin, particularly against *S. pyogenes*, *S. flexneri*, and *H. influenzae.* CAEO and LCEO produced weaker, strain-dependent effects. The combined synergy score and clustering analysis effectively distinguished synergistic from non-synergistic interactions, highlighting bacterial groups with higher susceptibility. These findings identify LNEO as a promising natural antibiotic potentiator and demonstrate the value of integrated chemical–biological analysis for evaluating EO–antibiotic combinations. Molecular docking provides valuable exploratory insights into the potential of the identified high-abundance compounds to bind and interact with the microbial protein targets. This is supported by the strong binding affinities and hydrophobic interactions exercised in the ligand–protein complex formation.

## Figures and Tables

**Table 1:** GC/MS analysis results of the three EOs studied.

Compound Class	Identified Compounds	LRIc *	LRIr * [54]	*CAEO*	*LCEO*	*LNEO*
				Conc. %	Conc. %	Conc. %
**Monoterpene Hydrocarbons**	α-Pinene	930	936	10.41	1.93	2.95
	Camphene	950	952		0.54	1.33
	β-Pinene	970	977	7.88	1.39	2.8
	Sabinene	978	981		1.75	4.67
	β-Myrcene	990	994	1.73	0.88	6.07
	3-Carene	1020	1012			0.2
	α-Phellandrene	1025	1029	0.82		1.71
	Limonene	1031	1030	9.09	19.19	30.68
	β-Ocimene Z	1040	1038	1.53		
	1,3,8-p-Menthatriene	1045	1048	1.61		
	β-Ocimene E	1051	1048	1.18		5.73
***Total***				**34.61**	**25.69**	**56.14**
**Oxygenated Monoterpenes**	Eucalyptol	1035	1032	52.09	1.73	28.4
	Camphor	1045	1043			9.08
	β-Linalool	1100	1099	2.28	1.85	0.92
	Myrcenol	1110	1113	2.27		1.6
	trans-3-Caren-2-ol	1131	1133			2.66
	Verbenol	1146	1144		0.9	
	β-Citronellal	1158	1154		3.15	
	4-Terpineol	1164	1165	3.3		
	β-Citral	1215	1220	0.55	42.98	
	Bornyl acetate	1286	1284			0.5
***Total***				**60.49**	**68.42**	**43.16**
**Sesquiterpene Hydrocarbons**	Caryophyllene	1408	1406		1.57	
	α-Caryophyllene	1420	1422		0.41	
	β-Elemene	1438	1436		0.14	
	β-Farnesene	1445	1446	1.13		
	α-Farnesene	1455	1456			0.7
	Germacrene B	1538	1535		0.11	
***Total***				**1.13**	**2.23**	**0.70**
**Oxygenated Sesquiterpenes**	Caryophyllene oxide	1580	1571		0.2	
	Alloaromadendrene oxide	1644	1649		0.15	
***Total***					**0.36**	
**Acyclic Alkynes And Alcohols**	1,3,6-Heptatriene, 5-methyl-	1055	1050	1.18		
	1,9-Decadiyne	1258	1262	0.64		
	3-Nonyn-2-ol	1590	1586	0.24		
	3-Buten-2-ol, 2,3-dimethyl	1690	1685		0.18	
***Total***				**2.05**	**0.18**	
**Cyclic aldehydes and unsaturated cyclic compounds**	5-Hepten-2-one, 6-methyl	1333	1336		1.86	
	2,4,6-Trimethyl-3-cyclohexene-1-carboxaldehyde	1398	1400		1.28	
***Total***					**3.14**	
**Aromatic Compounds (Phenylpropenes and Ethers)**	Estragole	1380	1384	0.53		
	Eugenol methyl ether	1560	1555	1.18		
***Total***				**1.71**		

*LRIc—calculated linear retention index; *LRIr—literature linear retention index. Relative percentages represent normalised peak areas calculated from the total ion chromatogram, with the total peak area set to 100% for each EO.

**Table 2:** Molecular docking outcome of high abundant compounds in *Lauraceae* EO with skin microbial protein targets.

S/No	Ligands	Binding Energy (Kcal/Mol)						Amino Acids Involved in the Interaction					
		2WLA	3U2D	3UUE	5TZ1	6V7X	7EIP	2WLA	3U2D	3UUE	5TZ1	6V7X	7EIP
1	α-Pinene (CID: 6654)	−4.7	−5.0	−4.7	−6.1	−6.7	−5.2	NIR	Ile B: 86	Trp A: 229, Val A: 233, Phe A: 276	Phe A: 233	NIR	NIR
2	β-Pinene (CID: 14896)	−4.6	−4.9	−4.6	−5.9	−6.6	−5.3	NIR	NIR	Trp A: 229, Val A: 233, Phe A: 276	Trp B: 54, Ile A: 55, Ala A: 62	NIR	NIR
3	Limonene (CID: 22311)	−4.9	−5.5	−5.2	−6.6	−7.6	−5.1	Ala A: 106, Arg A: 120	Ile B: 51, Ile B: 86, Leu B: 103, Ile B: 175	Trp A: 248, Tyr A: 250	Leu A: 87, Leu A: 88, Lys A: 90, Phe A: 233, Phe A: 380, Tyr A: 401	Leu B: 36, Tyr B: 56, Tyr B: 64, Trp B: 88	Phe A: 240, Leu A: 422, Lys A: 465, Leu A: 469
4	Ocimene (CID: 5281553)	−4.4	−5.5	−4.7	−5.8	−6.6	−5.1	Val A: 13, Lys A: 29, Ala A: 30, Leu A: 83	Ile B: 51, Ile B: 86, Leu B: 103, Ile B: 175	Leu A: 110, Trp A: 229, Val A: 233, Phe A: 276	Lys A: 90, Met A: 92, Phe A: 233, His A: 377, Phe A: 380, Tyr A: 401	Tyr B: 64, Val B: 76, Trp B: 88, Phe B: 101, Ala B: 105, Leu B: 110, Ala B: 127	Leu A: 293, Val A: 334, Leu A: 342
5	β-Myrcene (CID: 31253)	−4.3	−5.2	−4.5	−5.9	−5.8	−4.9	Val A: 49, Ala A: 106, Lys A: 112, His A: 117, Arg A: 120	Ile B: 51, Val B: 79, Ile B: 86, Ile B: 175	Leu A: 110, Trp A: 229, Val A: 233, Phe A: 276	Ala A: 117, Pro A: 230, Phe A: 233, His A: 377, Phe A: 380, Tyr A: 401	Leu B: 36, Leu B: 40, Tyr B: 47, Tyr B: 64, Val B: 76, Leu B: 125, Ala B: 127	Val A: 334, Leu A: 342
6	Eucalyptol (CID: 2758)	−4.7	−4.9	−4.5	−5.8	−6.5	−5.0	NIR	NIR	NIR	Trp B: 54	NIR	NIR
7	Camphor (CID: 2537)	−4.3	−4.7	−4.4	−6.2	−6.8	−4.8	NIR	NIR	NIR	Trp B: 54	NIR	NIR
8	Copaene (CID: 12303902)	−5.7	−6.5	−5.6	−8.0	−6.5	−6.3	Tyr A: 10, Ala A: 11	Ile B: 86	Trp A: 116, Leu A: 238	Phe B: 52, Trp B: 54, Ala A: 62	Leu A: 10, Leu C: 10, Leu A: 53, Ala A: 56, Leu A: 57,	Ala A:292, Leu A: 342
9	α-Bergamotene (CID: 86608)	−4.6	−6.8	−5.1	−7.7	−8.0	−6.1	Leu A: 103, Tyr A: 127	Ile B: 51, Ile B: 86, Ile B: 175	Trp A: 229, Val A: 233, Phe A: 276	Leu A: 87, Ala A: 117, Pro A: 230, Phe A: 233, Phe A: 380, Tyr A: 401	Leu B: 36, Ile B: 52, Tyr B: 56, Trp B: 60, Tyr B: 64, Ala B: 70, Val B: 76, Phe B: 101, Ala B: 127	Ala A: 292, Leu A: 293, Val A: 334, Leu A: 342
10	Caryophyllene (CID: 5281515)	−5.7	−5.9	−5.8	−7.6	−7.2	−5.9	NIR	NIR	Trp A: 229, Val A: 233	NIR	NIR	NIR
11	α-Caryophyllene (CID: 5281520)	−5.4	−6.1	−6.1	−7.7	−6.4	−5.8	NIR	NIR	NIR	Trp B: 54, Ala A: 62	Trp C: 45	NIR
12	Germacrene D (CID: 5317570)	−5.8	−7.1	−6.2	−7.9	−6.7	−6.3	Ala A: 106, Ala A:119, Arg A: 120	Ile B: 86	Tyr A: 250	Trp B: 54, Ala A: 62	Phe C: 19	Phe A: 240
13	α-Citral (CID: 638011)	−4.6	−5.4	−4.8	−6.2	−6.6	−4.9	Val A: 13, Ala A: 30, Ala A: 89	Ile A: 51, Ile A: 86, Ile A: 175	Val A: 266, Leu A: 270	Tyr A: 118, Leu A: 121, Tyr A: 132, Leu A: 376, His A: 377. Ser A: 378, Met A: 508	Leu B: 36, Tyr B: 56, Tyr B: 64, Leu B: 110	Ala A: 278, Leu A: 286, Arg A: 306, Leu A: 342
14	β-Citral (CID: 643779)	−4.5	−5.7	−4.4	−5.3	−6.1	−5.2	Ala A: 30, Asn A: 33, Ala A: 89, Tyr A: 91	Ile A: 51, Val A: 79, Gly A: 85, Ile A: 86, Ile A: 175	Trp A: 248, Tyr A: 250, Lys A: 258, Tyr A: 260	Ile B: 197, Phe B: 213, Ala B:218, Tyr B: 221	Leu B: 36, Leu B: 40, Tyr B: 47, Ile B: 52, Tyr B: 64, Ala B: 70, Val B: 76	Tyr A: 392, Phe A: 428, Ser A: 431, Leu A: 449

NIR; No Interacting Residue.

## Data Availability

The original contributions presented in this study are included in the article/Supplementary Material. Further inquiries can be directed to the corresponding author.

## Supplementary Materials

The following supporting information can be downloaded at: https://www.mdpi.com/article/10.3390/ijms27031447/s1.
