# Supplementary material for: Chemical Profiling, Ampicillin Interaction Patterns, and Exploratory Molecular Docking of Lauraceae Essential Oils
Source: Int J Mol Sci. 2026 Jan 31;27(3):1447. doi: 10.3390/ijms27031447 (PMC12897871; doi:10.3390/ijms27031447)
Supplement: Supplementary file 1 [file ijms-27-01447-s001.zip › ijms-4081191-supplementary.pdf]

Figure S1. GC–MS total ion chromatogram (TIC) of *Cryptocarya agathophylla* essential oil (CAEO).

The chromatogram shows the separation of volatile constituents identified by mass spectral matching and retention index comparison. Peak numbering corresponds to the compound identification listed in Table S1.

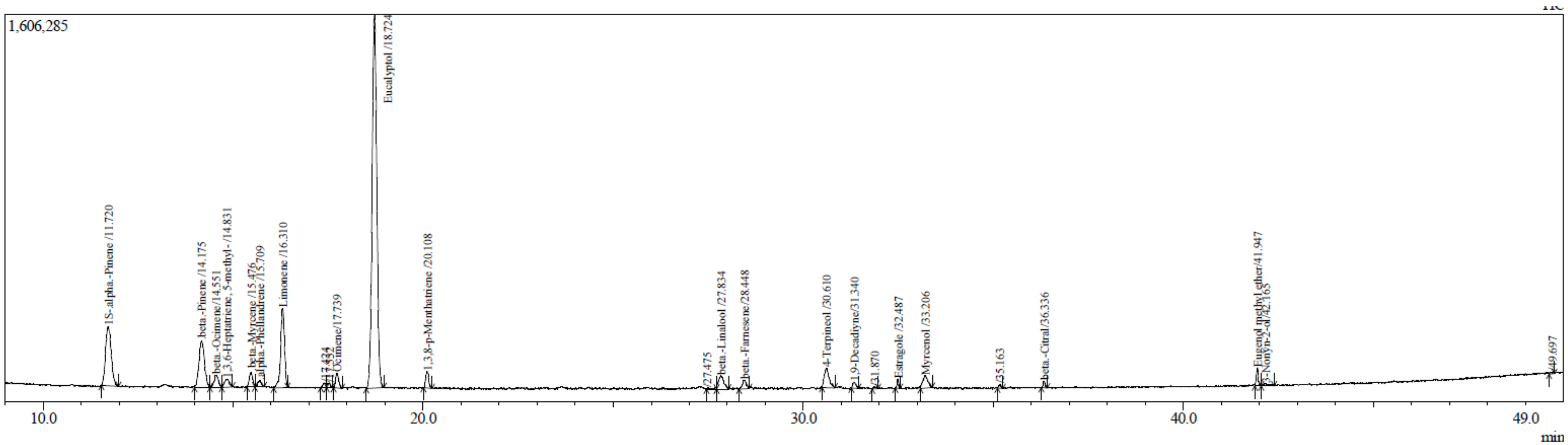

Table S1. Quantitative GC–MS composition of *Cryptocarya agathophylla* essential oil (CAEO).

The table presents the volatile compounds identified in *Cryptocarya agathophylla* essential oil by GC–MS analysis, including retention time (RT), peak area, peak height, and relative percentage composition.

ID	Compound name	m/z	Area	Height	Conc (%)
1	α-Pinene	TIC	2,636,743	249,914	10.412
2	β-Pinene	TIC	1,996,428	191,953	7.883
3	β-Ocimene	TIC	386,677	51,072	1.527
4	1,3,6-Heptatriene, 5-methyl-	TIC	299,656	34,201	1.183
5	β-Myrcene	TIC	438,794	65,703	1.733
6	α-Phellandrene	TIC	207,827	30,845	0.821

7	Limonene	TIC	2,302,200	330,375	9.091
8	Ocimene	TIC	389,641	63,613	1.539
9	Eucalyptol (1,8-cineole)	TIC	13,192,350	1,550,807	52.094
10	1,3,8-p-Menthatriene	TIC	406,952	72,254	1.607
11	β-Linalool	TIC	576,850	55,145	2.278
12	β-Farnesene	TIC	286,171	37,329	1.130
13	4-Terpineol	TIC	836,481	84,662	3.303
14	1,9-Decadiyne	TIC	160,865	27,627	0.635
15	Estragole	TIC	134,720	39,524	0.532
16	Myrcenol	TIC	573,684	54,254	2.265
17	β-Citral	TIC	139,909	31,218	0.552
18	Eugenol methyl ether	TIC	298,537	77,283	1.179
19	3-Nonyn-2-ol	TIC	59,717	10,057	0.236

Figure S2. GC–MS total ion chromatogram (TIC) of *Litsea cubeba* essential oil (CAEO). The chromatogram shows the separation of volatile constituents identified by mass spectral matching and retention index comparison. Peak numbering corresponds to the compound identification listed in Table S2.

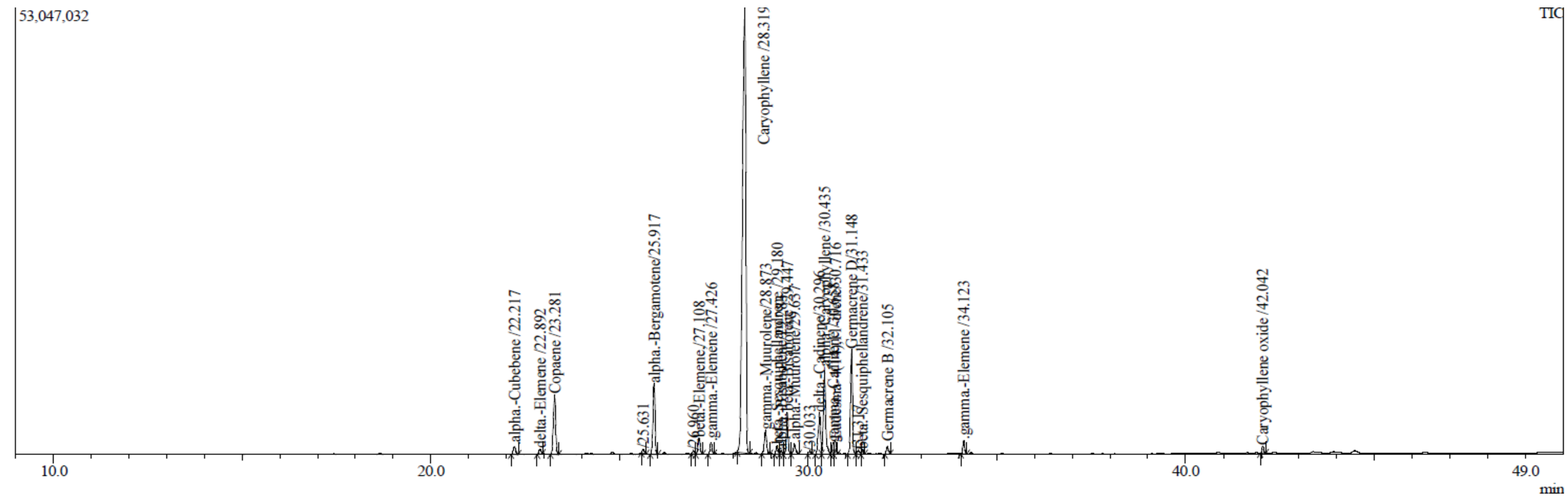

Table S2. Quantitative GC–MS composition of *Litsea cubeba* essential oil (CAEO).

The table presents the volatile compounds identified in *Litsea cubeba* essential oil by GC–MS analysis, including retention time (RT), peak area, peak height, and relative percentage composition.

ID	Compound name	m/z	Area	Height	Conc. (%)
1	α-Cubebene	TIC	4,461,546	951,423	0.747
2	δ-Elemene	TIC	3,323,161	641,671	0.557
3	Copaene	TIC	32,351,270	7,162,524	5.418
4	α-Bergamotene	TIC	32,882,569	8,591,572	5.507
5	β-Elemene	TIC	8,221,330	2,003,278	1.377
6	γ-Elemene	TIC	5,608,510	1,451,123	0.939
7	Caryophyllene (β-caryophyllene)	TIC	33,694,784	5,334,215	56.428
8	γ-Muurolene	TIC	12,564,539	2,912,421	2.104
9	β-Sesquiphellandrene	TIC	3,901,458	1,048,323	0.653

10	α-Bisabolene	TIC	1,232,491	405,291	0.206
11	β-Bisabolene	TIC	16,047,910	5,163,011	2.688
12	α-Muurolene	TIC	5,239,059	1,230,168	0.877
13	δ-Cadinene	TIC	18,932,034	5,007,573	3.171
14	α-Caryophyllene (α-humulene)	TIC	39,248,020	9,958,959	6.573
15	γ-Cadinene	TIC	4,883,670	1,262,983	0.818
16	Eudesma-4(14),11-diene	TIC	8,465,924	1,485,151	1.418
17	Germacrene D	TIC	48,000,751	12,603,043	8.039
18	β-Sesquiphellandrene	TIC	2,290,425	581,082	0.384
19	Germacrene B	TIC	3,716,540	1,009,777	0.622
20	γ-Elemene	TIC	5,688,915	1,667,465	0.953
21	Caryophyllene oxide	TIC	3,116,412	927,561	0.522

Figure S3. GC–MS total ion chromatogram (TIC) of *Laurus nobilis* essential oil (CAEO). The chromatogram shows the separation of volatile constituents identified by mass spectral matching and retention index comparison. Peak numbering corresponds to the compound identification listed in Table S1.

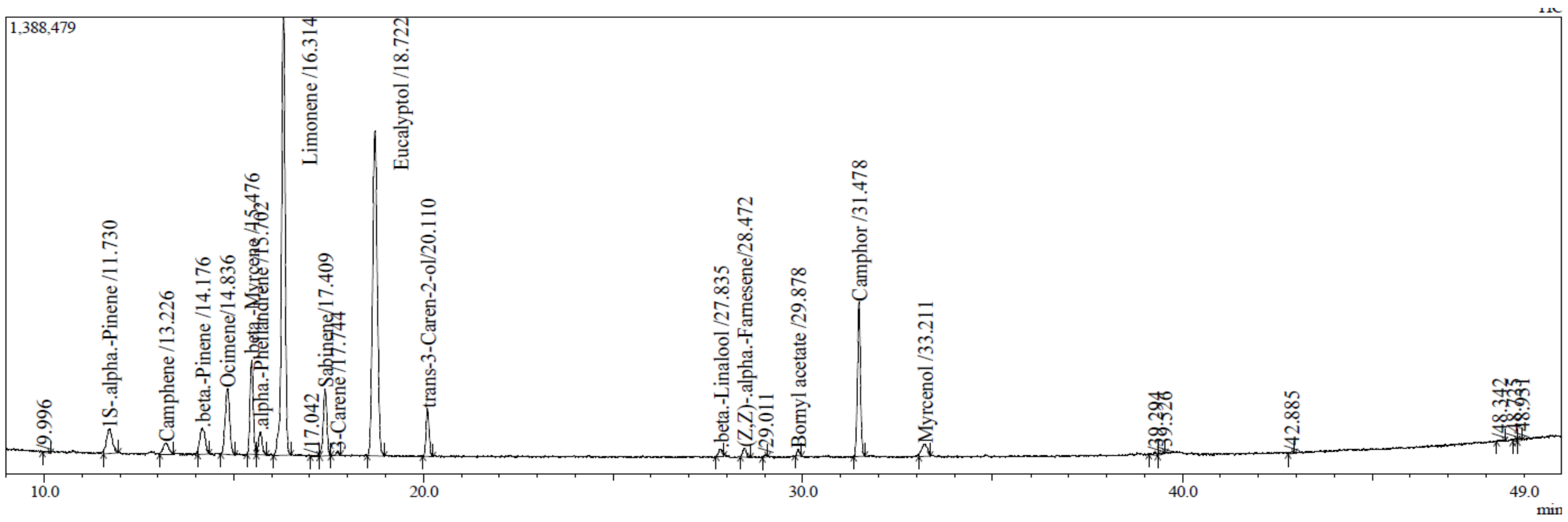

Table S3. Quantitative GC–MS composition of *Laurus nobilis* essential oil (CAEO).

The table presents the volatile compounds identified in *Laurus nobilis* essential oil by GC–MS analysis, including retention time (RT), peak area, peak height, and relative percentage composition.

ID	Compound name	m/z	Area	Height	Relative content (%)
1	α-Pinene	TIC	869,697	76,187	2.950
2	Camphene	TIC	392,920	35,846	1.333
3	β-Pinene	TIC	824,951	81,263	2.798
4	Ocimene	TIC	1,689,818	200,538	5.732
5	β-Myrcene	TIC	1,789,893	289,018	6.071
6	α-Phellandrene	TIC	503,985	71,644	1.709
7	Limonene	TIC	9,044,200	1,332,349	30.677
8	Sabinene	TIC	1,376,045	203,240	4.667
9	3-Carene	TIC	58,977	12,330	0.200
10	Eucalyptol (1,8-cineole)	TIC	8,373,856	984,852	28.403

11	trans-3-Caren-2-ol	TIC	782,904	145,079	2.656
12	β-Linalool	TIC	270,270	26,251	0.917
13	(Z,Z)-α-Farnesene	TIC	206,730	28,671	0.701
14	Bornyl acetate	TIC	148,353	24,549	0.503
15	Camphor	TIC	2,678,093	471,344	9.084
16	Myrcenol	TIC	471,580	39,695	1.600

Table S4. OD- and inhibition-based growth suppression profiles of *Lauraceae* essential oils applied alone or in combination with ampicillin on Gram-positive strains. Inhibition values were calculated from optical density measurements and are provided exclusively for qualitative–comparative analysis of antimicrobial trends and sub-MIC potentiation effects.

EO / Conc. (µL/mL)	*S. pyogenes* EO (%)	*S. pyogenes* EO + Amp (%)	*S. aureus* EO (%)	*S. aureus* EO + Amp (%)	*L. monocytogenes* EO (%)	*L. monocytogenes* EO + Amp (%)	*B. cereus* EO (%)	*B. cereus* EO + Amp (%)	*C. perfringens* EO (%)	*C. perfringens* EO + Amp (%)
CAEO 0.015	-11.83	-3.91	-8.58	1.76	-11.84	16.09	-31.17	5.12	-3.09	-8.08
CAEO 0.03	-10.01	6.37	-6.37	2.02	-8.99	30.64	-9.74	10.81	3.09	44.95
CAEO 0.06	15.93	7.53	8.40	15.84	-6.03	46.23	7.53	35.72	28.38	79.67
CAEO 0.125	29.17	14.82	9.59	22.39	18.91	62.51	11.39	42.66	33.29	80.18
CAEO 0.25	32.56	31.12	14.70	46.69	26.55	77.29	14.88	47.90	75.02	82.58
CAEO 0.5	39.22	49.45	19.03	51.51	40.97	79.14	45.09	52.67	82.74	83.84
CAEO 1.0	47.69	55.97	51.50	76.57	53.37	79.95	52.96	72.35	83.77	84.09
LCEO 0.015	-24.58	-17.85	-5.62	3.71	-4.23	7.66	-6.84	-13.96	-16.47	-15.15
LCEO 0.03	-22.06	-16.79	4.43	7.20	5.84	12.74	-6.50	-12.36	-14.57	3.66
LCEO 0.06	-21.60	-16.47	7.69	9.72	6.10	14.24	-2.65	-11.79	-6.14	11.99
LCEO 0.125	-20.59	-14.99	12.80	11.41	6.59	14.24	-2.43	-9.40	-2.69	12.63

LCEO 0.25	-20.31	-2.64	14.66	12.60	7.27	14.93	-1.32	-7.32	8.47	15.78
LCEO 0.5	-20.13	-1.80	17.53	13.46	8.35	17.36	-0.88	-0.61	15.68	19.32
LCEO 1.0	-17.05	-0.11	19.78	21.27	10.67	18.17	0.55	1.93	20.11	22.60
LNEO 0.015	-5.29	83.10	-5.72	0.97	-3.30	-5.04	-10.80	-2.88	-6.65	-1.30
LNEO 0.03	-4.52	83.63	4.22	13.61	-2.10	1.08	-6.54	-0.72	-6.33	0.34
LNEO 0.06	-1.19	84.37	4.33	14.43	-1.54	3.73	-6.28	-0.34	-5.03	2.69
LNEO 0.125	-0.77	84.48	4.43	15.84	-0.26	6.89	-2.83	0.64	-3.68	5.47
LNEO 0.25	5.99	84.79	7.08	15.66	0.37	11.74	-1.54	0.49	-1.74	9.09
LNEO 0.5	6.41	85.22	7.22	16.70	3.15	13.39	-0.15	1.40	-1.31	9.72
LNEO 1.0	6.97	85.43	7.55	17.28	7.83	17.36	0.59	2.69	2.30	12.50

Negative inhibition values indicate growth stimulation relative to the untreated control.

Table S5. OD- and inhibition-based growth suppression profiles of *Lauraceae* essential oils applied alone or in combination with ampicillin on Gram-negative strains. Inhibition values were calculated from optical density measurements and are provided exclusively for qualitative–comparative analysis of antimicrobial trends and sub-MIC potentiation effects.

EO/Concentration (µL/mL)	*S. flexneri* EO (%)	*S. flexneri* EO + antibiotic (%)	*P. aeruginosa* EO (%)	*P. aeruginosa* EO + antibiotic (%)	*E. coli* EO (%)	*E. coli* EO + antibiotic (%)	*S. typhimurium* EO (%)	*S. typhimurium* EO + antibiotic (%)	*H. influenzae* EO (%)	*H. influenzae* EO + antibiotic (%)
CAEO 0.015	-30.11	-14.02	-4.33	-7.00	8.45	-3.41	6.74	2.32	-0.38	-1.77
CAEO 0.03	-15.14	-11.27	-1.84	-5.32	1.70	-5.97	1.94	2.41	0.71	2.39
CAEO 0.06	-8.75	-6.30	-1.52	-4.19	1.18	-7.87	0.81	2.29	1.01	3.16
CAEO 0.125	-6.00	-3.47	-1.19	-1.84	0.66	-8.64	0.74	3.36	1.15	6.16
CAEO 0.25	-4.85	-0.84	-0.87	-1.39	0.28	-9.17	0.22	3.93	3.97	8.07
CAEO 0.5	-4.43	12.40	0.76	-0.71	-0.26	-8.95	0.15	3.98	4.84	8.15
CAEO 1.0	-1.07	13.96	1.84	4.01	-0.66	-11.67	-0.12	4.13	6.05	8.40
LCEO 0.015	-18.62	-9.86	-2.38	-5.84	-1.35	-13.68	3.59	0.25	4.31	2.68
LCEO 0.03	6.90	-9.51	-1.62	-2.58	-0.66	-6.66	2.48	0.00	13.13	15.30

LCEO 0.06	12.20	1.53	-0.76	-1.57	-0.69	-4.74	2.19	0.32	14.71	15.47
LCEO 0.125	12.25	5.17	-0.54	-1.35	-0.09	2.67	2.06	0.50	14.71	16.05
LCEO 0.25	14.38	11.07	0.00	-0.56	0.17	7.03	1.55	0.57	15.00	16.37
LCEO 0.5	14.94	19.72	0.54	0.56	1.02	9.88	0.96	1.05	15.32	16.57
LCEO 1.0	15.53	21.02	1.52	1.12	2.03	10.88	0.98	1.77	15.56	16.74
LNEO 0.015	-1.93	44.03	2.71	0.49	-13.46	-4.46	78.66	-1.82	88.63	-0.17
LNEO 0.03	-1.77	47.41	7.14	2.06	-12.33	-3.28	81.07	-1.57	90.02	17.38
LNEO 0.06	-0.56	78.78	7.76	3.90	1.02	-0.92	81.17	-1.24	90.37	17.63
LNEO 0.125	-0.08	82.34	8.87	6.10	2.41	4.94	81.69	-0.95	90.73	18.17
LNEO 0.25	3.45	82.94	11.51	8.13	2.29	7.84	85.52	-0.65	90.73	18.71
LNEO 0.5	3.42	83.75	12.41	10.97	2.74	10.62	85.84	-0.20	91.09	18.65
LNEO 1.0	4.43	83.64	13.13	13.93	3.86	13.76	87.09	1.22	91.30	18.59

Negative inhibition values indicate growth stimulation relative to the untreated control.

Table S6. Minimum inhibitory concentrations (MICs) of *Lauraceae* essential oils alone and in combination with ampicillin. MIC was defined as the lowest concentration inhibiting visible bacterial growth after incubation (≥90% inhibition). When no concentration achieved this threshold, MIC was recorded as not reached (> maximum tested concentration)

Strain	EO	MIC EO alone (µL/mL)	MIC EO + antibiotic (µL/mL)	Interaction (MIC-based)
S. pyogenes	CAEO	> max tested	> max tested	None
	LCEO	> max tested	> max tested	None
	LNEO	0.03	0.015	Synergy (MIC reduction)
S. aureus	CAEO	> max tested	> max tested	None
	LCEO	> max tested	> max tested	None
	LNEO	> max tested	> max tested	None
L. monocytogenes	CAEO	> max tested	> max tested	None
	LCEO	> max tested	> max tested	None
	LNEO	> max tested	> max tested	None
S. flexneri	CAEO	> max tested	> max tested	None
	LCEO	> max tested	> max tested	None

	LNEO	0.03	0.015	Synergy (MIC reduction)
P. aeruginosa	CAEO	> max tested	> max tested	None
	LCEO	> max tested	> max tested	None
	LNEO	> max tested	> max tested	None
E. coli	CAEO	> max tested	> max tested	None
	LCEO	> max tested	> max tested	None
	LNEO	> max tested	> max tested	None
S. typhimurium	CAEO	> max tested	> max tested	None
	LCEO	> max tested	> max tested	None
	LNEO	> max tested	> max tested	None
H. influenzae	CAEO	> max tested	> max tested	None
	LCEO	> max tested	> max tested	None
	LNEO	0.03	0.015	Synergy (MIC reduction)
